# Transitional Care for Young People with Movement Disorders: Consensus‐Based Recommendations from the MDS Task Force on Pediatrics

**DOI:** 10.1002/mdc3.13728

**Published:** 2023-04-04

**Authors:** Tamara Pringsheim, Amit Batla, Ali Shalash, Jitendra Kumar Sahu, Carlos Cosentino, Darius Ebrahimi‐Fakhari, Jennifer Friedman, Jean‐Pierre Lin, Jonathan Mink, Alexander Munchau, Daniela Munoz, Nardo Nardocci, Belen Perez‐Dueñas, Zomer Sardar, Chahnez Triki, Hilla Ben‐Pazi, Laura Silveira‐Moriyama, Monica Troncoso‐Schifferli, Kyoko Hoshino, Russell C. Dale, Victor S.C. Fung, Manju A. Kurian, Emmanuel Roze

**Affiliations:** ^1^ Department of Clinical Neurosciences Psychiatry, Pediatrics and Community Health Sciences, University of Calgary Calgary AB Canada; ^2^ Department of Clinical and Movement Neuroscience UCL Queen Square Institute of Neurology London UK; ^3^ Department of Neurology Faculty of medicine, Ain Shams Univeristy Cairo Egypt; ^4^ Pediatric Neurology Unit, Postgraduate Institute of Medical Education and Research Chandigarh India; ^5^ Department of Neurodegenerative Diseases Instituto Nacional de Ciencias Neurologicas and School of Medicine, Universidad Nacional Mayor de San Marcos Lima Peru; ^6^ Department of Neurology Boston Children's Hospital, Harvard Medical School Boston MA USA; ^7^ Departments of Neurosciences and Pediatrics UC San Diego San Diego CA USA; ^8^ Children's Neurosciences, Complex Motor Disorders Service (CMDS) Evelina London Children's Hospital, Guy's and St Thomas’ NHS Foundation Trust (GSTT), and Women and Children's Health Institute Faculty of Life Sciences & Medicine, Kings Health Partners, King's College London London UK; ^9^ Department of Neurology University of Rochester Rochester NY USA; ^10^ Institute of Systems Motor Science, University of Lübeck Lübeck Germany; ^11^ Department of Paediatric Neurology San Borja Arriaran Hospital. University of Chile Santiago Chile; ^12^ Pediatric Neuroscience Department Fondazione IRCCS Istituto Neurologico “C Besta” Milan Italy; ^13^ Department of Pediatric Neurology Hospital Universitari Vall d'Hebrón, Universitat Autònoma de Barcelona. Centre for Biomedical Research of Rare Diseases (CIBERER), ISCIII Madrid Spain; ^14^ FCPS, Department of Neurology Columbia University Irving Medical Center/New York Presbyterian Hospital New York NY USA; ^15^ Department of child neurology Hedi Chaker Hospital, LR10ES15, Sfax Medical School, University of Sfax Tunisia Sfax Tunisia; ^16^ Movement Disorders Clinic, Assuta Ashdod Ahdod Israel; ^17^ Department of Neurology University of Campinas (UNICAMP) Campinas Brazil; ^18^ Segawa Memorial Neurological Clinic for Children Tokyo Japan; ^19^ Children's Hospital at Westmead Clinical School, University of Sydney Sydney NSW Australia; ^20^ Movement Disorders Unit, Department of Neurology Westmead Hospital & Sydney Medical School, University of Sydney Sydney NSW Australia; ^21^ Developmental Neurosciences Zayed Centre for Research into Rare Disease in Children, GOS‐Institute of Child Health, UCL London UK; ^22^ Sorbonne University Paris Brain Institute, Assistance Publique—Hôpitaux de Paris, DMU Neurosciences Paris France

## Abstract

**Background:**

The International Parkinson and Movement Disorders Society (MDS) set up a working group on pediatric movement disorders (MDS Task Force on Pediatrics) to generate recommendations to guide the transition process from pediatrics to adult health care systems in patients with childhood‐onset movement disorders.

**Methods:**

To develop recommendations for transitional care for childhood onset movement disorders, we used a formal consensus development process, using a multi‐round, web‐based Delphi survey. The Delphi survey was based on the results of the scoping review of the literature and the results of a survey of MDS members on transition practices. Through iterative discussions, we generated the recommendations included in the survey. The MDS Task Force on Pediatrics were the voting members for the Delphi survey. The task force members comprise 23 child and adult neurologists with expertise in the field of movement disorders and from all regions of the world.

**Results:**

Fifteen recommendations divided across four different areas were made pertaining to: (1) team composition and structure, (2) planning and readiness, (3) goals of care, and (4) administration and research. All recommendations achieved consensus with a median score of 7 or greater.

**Conclusion:**

Recommendations on providing transitional care for patients with childhood onset movement disorders are provided. Nevertheless several challenges remain in the implementation of these recommendations, related to health infrastructure and the distribution of health resources, and the availability of knowledgeable and interested practitioners. Research on the influence of transitional care programs on outcomes in childhood onset movement disorders is much needed.

Pediatric movement disorders (PMDs) is a growing subspecialty, encompassing a variety of mostly hyperkinetic disorders. It is a diverse scope of practice, with the most common symptoms encountered including tics, stereotypies and tremor, with a growing list of recently identified genetic and acquired disorders producing overlapping symptoms of paroxysmal or progressive dystonia, chorea, myoclonus, ataxia, spastic paraparesis and Parkinsonism. Children with PMDs are increasingly seen by subspecialists in movement disorders given their complexity, who may now offer a number of specialized therapies. Children with PMDs are surviving into adulthood and ultimately require transition to adult services. In general, adolescents transitioning to adult healthcare have a greater risk of poor health outcomes. Transition comes with challenges, due to the nature of pediatric compared to adult services. Pediatric services are typically family focused, with multidisciplinary teams providing care in a single setting, compared to the more individual focus of adult care, with non‐physician services often provided in a less integrated fashion. In recognition of the challenges in providing care to this population, the International Parkinson and Movement Disorders Society (MDS) set up a working group (MDS Task Force on Pediatrics) to generate recommendations on the transition process to adult health care in patients with PMDs.

## Methods

We used a formal consensus development process, using a multi‐round, web‐based Delphi survey. The Delphi method uses an anonymous voting system allowing an equal opportunity to express ideas and opinions. It provides more time to reflect on questions and make changes, using an iterative process that allows recommendations to be refined.

The Delphi survey was developed in English by five of the authors (T.P., E.R., J.S., A.B., A.S.) based on the results of our scoping review^1^ on this topic, and the survey of MDS members on transition practices.^2^ The scoping review identified transitional care issues and transitional care models for youth with chronic neurological conditions. Using this information as the foundation, we generated the recommendations included in the survey through iterative discussions among the five authors. The MDS Task Force on Pediatrics were the voting members for the Delphi survey. The task force members comprise 23 child and adult neurologists with expertise in the field of movement disorders and from all regions of the world.

The survey was disseminated to task force members using the Survey Monkey platform. All recommendations were supported by rationale statements which provide supporting information for recommendations and appropriate citations. All recommendations were rated by panelists on a 9‐point Likert scale, with a score of 1 representing “inappropriate” and 9 representing “appropriate.” The research team reviewed responses, calculating median results and incorporating narrative comments provided by panelists. Our established threshold for recommendation appropriateness was a median score of ≥7, and inappropriate if the median score was ≤5. If the median score fell between 5 and 7, the recommendation was discussed for exclusion or revision with revoting. The recommendation was determined to generate disagreement if one third of the participants rated the item <5. All voting members of the Delphi survey could provide written comments so that statements could be altered if consensus was not achieved.

## Results

All 23 members of the MDS Task Force on Pediatrics were invited to participate in the anonymous Delphi process, of whom 19 completed the survey. Panel members were from all five continents and represented 17 countries. Table 1 presents the median and interquartile ranges for the recommendations in the first round of the Delphi process. As all recommendations achieved consensus with a median score of 7 or greater, and no recommendations had more than one third of participants scoring less than 5, a second round of voting was not required.

**TABLE 1:** Median and interquartile ranges for the recommendations in the Delphi process

Recommendation	Median Score and Interquartile Range
1.1	9 (7.75, 9)
1.2	9 (8, 9)
1.3	9 (8, 9)
1.4	9 (7, 9)
2.1	9 (8, 9)
2.2	9 (8, 9)
2.3	8 (7.25, 9)
3.1	9 (8, 9)
3.2	9 (8, 9)
3.3	9 (8, 9)
3.4	8 (7, 9)
3.5	9 (9, 9)
4.1	9 (8, 9)
4.2	9 (8, 9)
4.3	9 (8, 9)

## Recommendations

### Transitional Care: Team Composition and Structure

#### Recommendation 1.1

A transitional care service for young people with movement disorders should comprise a multidisciplinary team. Based on personalized transition planning, this team may be composed of both pediatric and adult care providers, including a transition coordinator, physicians, social workers, nurses, physical, occupational and mental health therapists and other allied health professionals.

#### Rationale for Recommendation 1.1

Multidisciplinary team transition models have been successfully applied for children with chronic neurological conditions including movement disorders. The survey conducted by the MDS task force showed that current transition services follow a multidisciplinary approach.

#### Recommendation 1.2

The adult physician assuming care of the transitioning patient should have necessary knowledge about the young person, expertise in neurological disorders starting in infancy/childhood and be attentive to the comprehensive care needs of young adults. They should have awareness and knowledge of transitional care issues including intellectual disability and ability to participate in treatment planning, especially in people with movement disorders.

#### Rationale for Recommendation 1.2

In general, adult physicians are reported as having poor knowledge of neurological disease starting in childhood, including intellectual disability^3^ and cerebral palsy.^4^ This may not apply to all neurologists specializing in movement disorders. Pediatricians are considered more attentive to the often complex care needs of young adults, with pediatric hospitals providing more services and resources.^4^ Inadequate care and a disparate health care environment and culture was described in the adult system.^5^, ^6^, ^7^ If the adult lead of transition service had better awareness and knowledge of transitional care issues in people with movement disorders, this can help minimize the disparity in the healthcare environment during the transition process.

#### Recommendation 1.3

A transition coordinator should be assigned to each transitioning patient, who should take charge of documentation, sharing medical records and communication with patients and families.

#### Rationale for Recommendation 1.3

Communication and documentation can be challenging in complex cases. A transition coordinator can help keep communication focused and ensure documentation. Usually a dedicated transition nurse can take this role. The survey results showed that some teams already used this approach and some other studies have highlighted the importance of transition coordinators in improving health outcomes. The beneficial features of transition services include a written transition plan, appropriate parental involvement, and involvement of a transition coordinator.^8^

#### Recommendation 1.4

The transition service can look at adopting examples from best practice in other medical conditions like diabetes and neurological disorders such as epilepsy that have been providing transition services within the same country, region or hospital.

#### Rationale for Recommendation 1.4

There is very limited evidence on running a successful transition service for movement disorders, however, some successful models of transitional care in other neurology specialties exist and these have already identified local and global issues. Liaising with local teams running transition services for other conditions might give better insights and best practice examples.

### Transitional Care: Planning and Readiness

#### Recommendation 2.1

A transitional planning period should occur, during which time transition readiness is assessed, the young person receives training in health self‐efficacy, and a formal transition plan is developed and documented.

#### Rationale for Recommendation 2.1

Inadequate preparation of patients transitioning to adult services has been highlighted by several studies of youth with complex neurological conditions, intellectual impairment, and cerebral palsy.^3^, ^4^, ^6^ A study of health outcomes of young people with diabetes, autism and cerebral palsy found that those with greater health self‐efficacy were more satisfied with services related to transition from pediatric to adult health care.^9^ Parents of children with intellectual disability recommend the use of a transition planning document which includes all necessary steps for the transition, including legal and financial steps, and contact details for all involved professionals and organizations.^3^

#### Recommendation 2.2

The timing and approach of transition should be flexible and made collaboratively by the pediatric care providers, transitional care team, patient and family. Evaluation of transition readiness should factor in the individual needs of the patient and their caregivers, their disease stage, and the presence of cognitive and behavioral problems, as well as any systemic disease features, rather than be based on age, *per se*.

#### Rationale for Recommendation 2.2

A qualitative study with parents of children with intellectual disabilities found that the decision to transition from pediatric to adult care at 18 years of age felt arbitrary to parents, as their young adult would always remain a “child” due to their intellectual impairment. Parents perceived that the instability of their young adults’ health status was not truly appreciated by the pediatric services during the process of transition, which in several cases lead to the need for acute hospitalization in the adult setting.^6^ Young people with cognitive and behavioral conditions experience greater challenges in preparing for transition to adult health care. In a study of youth with type 1 diabetes, Turner syndrome, spina bifida and autism, significant differences in transition readiness were found according to diagnosis. While youth with type 1 diabetes did not differ from youth without chronic medical conditions, youth with Turner syndrome, spina bifida and autism had significantly lower transition readiness, with youth with autism having the lowest transition readiness scores. Higher transition readiness scores were observed in youth who were older, lived in two parent households, and had higher levels of health literacy.^10^

#### Recommendation 2.3

A dedicated transition planning visit, including the patient, their family and the transitional care team should occur pre‐transition. The case history, problem list, transitional goals and transitional plan should be first reviewed in depth during the transition planning visit and again during the period of overlapping care.

#### Rationale for Recommendation 2.3

Parents of children with chronic complex neurological conditions with intellectual impairment report that they received little preparation for the transition from pediatric to adult health care and experienced an abrupt termination of pediatric services without appropriate assurance of future care in the adult setting. Parents perceived very limited coordination of the transition and lack of communication among health care professionals in the months before their child turned 18.^6^ Parents of children with intellectual disability report that although their child is the center of the transition, the parents experience the effects of the transition and its different stages. However, they are often ignored in the transition process.^3^ Young adults with intellectual impairment require significant guidance from their parents for decision making related to their care, and this reliance on parents is unlikely to change over time. In a study of young people with diabetes, autism and cerebral palsy, there were significant positive associations between appropriate parental involvement (but with changing responsibilities) and satisfaction with services during the transition from pediatric to adult health services, as well as overall wellbeing during this period. This study also found that meeting the adult care team before transfer was significantly associated with autonomy in healthcare appointments.^9^

### Transitional Care: Goals of Care

#### Recommendation 3.1

Every youth with a movement disorder should have a planned transition of care with the goal to improve individual health outcomes and avoid adverse health or psychological outcomes during this critical period.

#### Rationale for Recommendation 3.1

Most children with chronic movement disorders are regularly seen by a pediatric neurologist or a pediatrician and allied specialties. Integration into the adult healthcare system can be challenging, making a planned and standardized approach to transition desirable. Insufficient preparation, poor anticipation and lack of a formalized transition plan have been identified as factors preventing an optimal transition in patients with chronic neurological disorders.^6^, ^7^, ^11^ Transition planning instead was reported as a potential solution to improve the transition process^3^, ^8^ and early transition preparation and planning has been recommended by a neurology transition consensus panel.^12^

#### Recommendation 3.2a

The transitional care service should address the medical, cognitive, rehabilitation, emotional, and social needs of the transitioning youth and their family.

#### Recommendation 3.2b

Issues to be discussed during the transition process should ideally comprise the following, with some individual tailoring:

1. adolescent's neurological condition and any co‐occurring medical or psychiatric conditions.
2. current medications, level of benefit in the patient and potential side effects.
3. signs and symptoms of concern.
4. disease related comorbidities.
5. general health issues and wellbeing.
6. young person's knowledge of their disease and medication.
7. genetic counseling (where relevant).
8. effect of puberty on disease and medical treatment.
9. sexuality, pregnancy (where appropriate) and reproductive implications of the condition.
10. alcohol, smoking and substance use.
11. emotional/psychological concerns.
12. perceived stigmatization.
13. change in family dynamics with increasing patient maturity.
14. vocational issues.
15. driving.
16. ability to consent for medical treatment, legal and financial transactions.

#### Rationale for Recommendation 3.2 (including 2a and 2b)

From a developmental point of view, youth age implies significant changes in various domains of life.^13^ Childhood movement disorders encompass heterogeneous conditions with various comorbidities^14^, ^15^ implying distinct needs of the youth and her/his family, not only for medical care and rehabilitation but also in the domains of cognition, emotional and social well‐being. These needs should be addressed in a holistic manner and some important issues are often not addressed during the transition process, such as addiction problems, driving, sexuality or pregnancy/reproductive issues. A checklist of key issues to be potentially addressed depending on each youth situation would thus help the transition teams.

#### Recommendation 3.3

Special attention should be paid to guide the transitioning patient towards more autonomy (where possible) for health‐related issues, including self‐management training which takes into account the intellectual and behavioral functioning of each patient.

#### Rationale for Recommendation 3.3

Youth with chronic neurological disorders tend to not have the level of independence and knowledge about their disease that is expected in adult settings, as clearly identified in patients with epilepsy.^16^ In addition, cognitive deficits and behavioral disturbances represent major brakes on the development of transition readiness.^10^ Autonomy is considered as a major ethical principle in adult health care and can influence the care trajectory. With moving towards adulthood, the focus of the health care environment should thus gradually move from a family‐based approach towards more autonomy in adult health care system. It is mandatory to ensure continuity of care and adequate continuation of drugs. It is therefore crucial to guide youth towards more autonomy for health‐related issues taking into account the individual intellectual and behavioral abilities.

#### Recommendation 3.4

Emotional support should be provided to the patient and their family during the transition process by the transitional care team, including preparing and accompanying patients to their first visit in the adult setting

#### Rationale for Recommendation 3.4

Transition of care is a time of uncertainty associated with a heightened emotional response, whereas emotional needs are often neglected during the transition process.^4^, ^6^ A significant proportion of patients with ADHD and their parents reported a need to have someone to speak with regarding their emotional needs during the process of transition.^17^ Transition‐related factors that could influence the emotional well‐being of patients with neurological disorders during this period can comprise difficulties with the practical aspects of transition, a sense of abandonment, the combination of sadness at leaving a system with familiar health professionals and trusted relationships and fear of the unknown.^4^, ^6^, ^17^, ^18^ Special attention should thus be paid to the emotional support of the youth and parents during the transition to the adult care system.

#### Recommendation 3.5

Both the pediatric and adult care providers should use the transition process as an opportunity to reappraise the diagnosis and treatment of the patient

#### Rationale for Recommendation 3.5

For many patients presenting in early childhood, the diagnostic odyssey may have occurred several years prior to transition, and thus re‐appraising the diagnosis based on interim scientific advances is important to ensure diagnostic accuracy. A study focused on patients with a childhood‐onset movement disorder demonstrated that a multidisciplinary approach involving a team with a pediatrician, a movement disorders specialist, a metabolic disorder expert and a geneticist can promote a high diagnostic yield (34%) in formerly undiagnosed patients and also result in treatment adjustment with positive consequences in more than 40% of patients.^19^ In a study about a transitional care clinic in patients with childhood‐onset epilepsy, diagnosis was revised in 12% of patients and 55% received adjustment of the anti‐epileptic medication.^20^ The core medical component of transition process includes a review of history, examination findings along with investigations and discussion in a multidisciplinary manner. Therefore, it offers a unique opportunity to reappraise the diagnosis and treatment of the youth with childhood‐onset movement disorders.

### Transitional Care: Administration and Research

#### Recommendation 4.1

Financial resources for the transition care services should be provided for well‐organized continuous and satisfactory transitional care

#### Rationale for Recommendation 4.1

Establishing transition care services requires resources to cover costs of services setting and documentation, funded therapy and fees for clinicians, nurses, social workers. Lack of resources was identified as a barrier to successful and satisfactory transition care.^8^, ^11^, ^21^, ^22^ Moreover, lack of resources was more apparent in adult services, in contrast to pediatric hospitals and facilities.^4^, ^22^ Therefore, additional resources are required to establish continuous and satisfactory transition care services.

#### Recommendation 4.2

There should be a framework for assessment, feedback, and quality improvement for transitional care services

#### Rationale for Recommendation 4.2

Previous studies reported inconsistent practices and lack of transition plans, protocols, and outcomes, implying the need for evaluation of these transition care programs. Evaluation of transition practices identifies the challenges, limitations, outcomes and satisfaction of patients and their families.^11^, ^20^, ^22^, ^23^ Evaluating patient‐related outcomes including, health, educational, and vocational issues reflects the effectiveness of transition services,^24^ and provides an important metric in optimizing transitional care models. Additionally, regular assessment of transition care is essential to evaluate and adapt services to sustain family satisfaction and community provider satisfaction.^25^ Therefore, assessment of transition care services is warranted to evaluate patients' outcome, cost‐effectiveness, limitations, difficulties, and inappropriate practices.

#### Recommendation 4.3

Research is needed and should be encouraged to evaluate the different aspects of transitional issues and the outcome and cost effectiveness of transitional care programs in different populations all over the world

#### Rationale for Recommendation 4.3

Our recent review showed only 56 articles discussed transition care issues, and only two articles included patients with movement disorders. Most of these studies were from the USA, UK and Canada. Moreover, most transitional care studies were cross sectional with limited subjects’ number.^1^ Few studies reported on care models and evaluated their outcome.^25^, ^26^, ^27^ Additionally, there is limited data on the outcome of transition programs and their cost‐effectiveness. Therefore, more studies are required to determine the needs and outcomes specific to this clinical population.

## Discussion

When a migrant has no alternative but to leave his country, he/she can make substantial efforts to get ready for the journey, but the true adventure actually starts when setting foot on land in the new country. Adolescents with PMDs are migrants of care and the transition to adult medical care is a major milestone. Adult movement disorders departments should implement coordinated transitional care, considering the growing number of patients and the specific issues related to this age group and the transition of care. Based on our scoping review^1^ and international survey^2^ on transition practices and transition issues, we have proposed recommendations to help implement such a care program.

Our approach was based on data from the literature and field knowledge to optimize and standardize translational care practice. We use a structured expert consensus method, which is recommended to address complex healthcare issues and limits the influence of individual bias. The level of agreement for all recommendations was very high. Nevertheless, some limitations exist. First, consensus methods inherently contain risk of bias in the selection of panel participants. Our panel did not include people with lived experience with PMDs. However, our panel involved physicians from five continents with a variety of cultural origins and living standards. Many of the studies in the scoping review were qualitative studies with people undergoing transition, allowing their perspectives to be incorporated. Second, the needs of the target population have been poorly studied and this might have hampered our abilities to properly anticipate the optimal care pathway. Third, during the expert discussions, it appeared that, depending on the country, accessibility to transitional care programs can be restricted because resources or infrastructure are limited and/or because public or private health insurance does not cover related costs. Fourth, beyond the issue of optimization and standardization of transitional care practice, a major problem is likely the insufficient number of neurologists adequately trained to take care of young adults with PMDs. This is possibly due to lack of interest, insufficient opportunity for training, and the lack of training standards in this area. Finally, while the international survey we used as foundational information was sent to all members of the MDS, only 2% participated.

During the process, expert discussion revealed shortcomings in knowledge in the field and opened avenues for future research and education. (1) Studies are mandatory to clarify the specific needs and patient‐desired outcomes of youth with PMDs. We need further insight into what patients and families experience as good care, and what kind of harms are engendered when that standard is not met. We suggest focusing on motor disability and psychiatric comorbidities, which are frequently encountered in this context. Likewise, the level of perceived stigmatization and body image concerns should be investigated as they likely represent a significant determinant of quality of life in young people with visible movement disorders.^28^ (2) The influence of transitional care programs on population health and individuals is difficult to evaluate because we need validated tools to measure it and a definition of successful transition in movement disorders. This will allow proper investigation of cost‐effectiveness of translational care programs. (3) The success of transition depends on the availability and efficacy of the transition care model but also on the capacity of patients and families to manage this period and the related constraints. More information is needed to determine the individual and community factors influencing management capacity of patients and families. (4) We need to improve attractivity of transition care in general, and of transition care for PMDs in particular, as well as training in this field. Research should be done on how to integrate transition curricula from undergraduate to postgraduate levels.

## Author Roles

(1) Research project: A. Conception, B. Organization, C. Execution; (2) Statistical Analysis: A. Design, B. Execution, C. Review and Critique; (3) Manuscript Preparation: A. Writing of the first draft, B. Review and Critique.

T.P.: 1A, 1B, 1C, 2A, 2B, 2C, 3A

A.B.: 1B, 1C, 2C, 3A

A.S.: 1B, 1C, 2C, 3A

J.K.S.: 1B, 1C, 2C, 3A

C.C.: 2C, 3C

D.E.F.: 2C, 3C

J.F.: 2C, 3C

J.P.L.: 2C, 3C

J.M.: 2C, 3C

A.M.: 2C, 3C

D.M.: 2C, 3C

N.N.: 2C, 3C

B.P.‐D.: 2C, 3C

Z.S.: 2C, 3C

C.T.: 2C, 3C

H.B.‐P.: 2C, 3C

L.S.‐M.: 2C, 3C

M.T.‐S.: 2C, 3C

K.H.: 2C, 3C

R.C.D.: 2C, 3C

V.S.C.F.: 1A, 2C, 3B

M.A.K.: 1A, 1B, 2C, 3B

E.R.: 1A, 1B, 1C, 2A, 2B, 2C, 3A

## Disclosures

**Ethical Compliance Statement:** The authors confirm that the approval of an institutional review board was not required for this work. The authors confirm that patient consent was not required for this work. We confirm that we have read the Journal's position on issues involved in ethical publication and affirm that this work is consistent with those guidelines.

**Funding Sources and Conflicts of Interest:** This study was funded by the International Parkinson's and Movement Disorders Society. The authors have no financial disclosures or conflicts of interest concerning the research related to the manuscript.

**Financial Disclosures for Previous 12 months:** Tamara Pringsheim has received research support paid to her institution from the Public Health Agency of Canada, Alberta Health and the Owerko Center of Alberta Children's Hospital Research Institute. Amit Batla has received speaker honorarium from Ipsen Pharma and receives royalties from the book “Understanding Parkinsonism” (Jaypee brothers 2017). Jitendra Kumar Sahu has received honorarium from the *Indian Journal of Pediatrics* for working as a Section Editor. He also received research grant support paid to his institution from the Indian Council of Medical Research, New Delhi. Darius Ebrahimi‐Fakhari has received speaker honoraria from the Movement Disorder Society, publishing royalties from Cambridge University Press, and research grants from the NIH/NINDS, the CureAP4 Foundation, the CureSPG50 Foundation, the Spastic Paraplegia Foundation, the Tom‐Wahlig Foundation, the BPAN Warriors, the Boston Children's Hospital Office of Faculty Development, the Manton Center for Orphan Disease Research, and a joint research agreement with Astellas Pharmaceuticals Inc. JP Lin has received support from the Guy's and St Thomas Charity New Services and Innovation Grant G060708; the Dystonia Society UK Grants 01/2011 and 07/2013; Action Medical Research GN2097 and unrestricted educational grants from Medtronic Ltd. Jonathan Mink has received compensation for consulting with Biogen, Amicus, Taysha, and Polaryx. He receives research funding from Neurogene. He serves on Data and Safety Monitoring Boards for PTC and Passage Bio. He serves as a central rater for a clinical trial sponsored by TEVA and a clinical trial executive committee for Applied Therapeutics. He receives research funding from the NIH. Alexander Munchau has received honoraria for lectures from Takeda, support from the Deutsche Forschungsgemeinschaft (DFG: SFB 936, and FOR 2698), and the European Reference Network—Rare Neurological Diseases (ERN – RND; Project ID No 739510) and royalties for the book *Neurogenetics* (Oxford University Press). Hilla Ben‐Pazi, Recived grant form Israeli Innovation Authority and Women‐Tech grant from the EU. Laura Silveira‐Moriyama has received honorary for Teva, Ipsen and Roche‐FQM, has received support from SAS Brasil, and editorial fees for Brain and Behavior. Victor Fung receives a salary from NSW Health, has received unrestricted research grants from the Michael J. Fox Foundation, Abbvie and Merz, has been on Advisory Boards for Abbvie, Allergan, Ipsen, Merz, Seqirus, Stada, Teva and UCB, and receives royalties from Health Press Ltd. Emmanuel Roze received honorarium for speech from Orkyn, Aguettant, Elivie and for participating in an advisory board from Merz‐Pharma. He received research support from Merz‐Pharma, Orkyn, Elivie, Everpharma, Fondation Desmarest, AMADYS, ADCY5.org, Fonds de dotation Patrick Brou de Laurière, Agence Nationale de la Recherche, Dystonia Medical Research Foundation. All other authors have nothing to disclose.
